# Multiple work demands and early retirement intention in Germany: A cross-sectional and longitudinal analysis

**DOI:** 10.1007/s10433-026-00915-y

**Published:** 2026-03-08

**Authors:** Arthur Kaboth, Sophie-Charlotte Meyer

**Affiliations:** Federal Institute for Occupational Safety and Health, Friedrich-Henkel-Weg 1-25, 44149 Dortmund, Germany

**Keywords:** Working conditions, Physical demands, Psychosocial demands, Pension, Fixed-effects regression

## Abstract

The present study examines the association between (multiple) physical and psychosocial work demands and the preference for early retirement among older German employees. The analyses are based on data from five waves (2015–2023) of the BAuA-Working Time Survey, including 32,686 person-year observations. The dependent variable is a binary indicator reflecting preference for early retirement versus statutory or later retirement. Cross-sectional analyses apply linear probability models (LPM) per wave, while longitudinal effects are assessed using pooled OLS (POLS), random-effects (RE), and fixed-effects (FE) estimators. Robustness checks include controls for health and job satisfaction.

Cross-sectional models show positive and mostly significant associations between both physical and psychosocial work demands with early retirement preference. Psychosocial demands, particularly workload pressure and emotional demands, are consistently strong predictors. In longitudinal FE analysis, multiple psychosocial work demands remain meaningful and significant.

The findings underscore the importance of multiple simultaneous psychosocial job demands, rather than only focusing on isolated working conditions, in shaping early retirement preferences. Despite modest coefficient sizes, these results based on retirement intentions (not actual exit decisions) highlight the necessity of a life-course perspective and targeted interventions to promote sustainable working lives.

**Supplementary Information:**

The online version contains supplementary material available at 10.1007/s10433-026-00915-y.

## Introduction

Over the next 10 to 15 years, the age structure of the workforce in Germany and across Europe will undergo substantial demographic shifts. With the exit of the so-called Baby Boomer generation—those born between 1955 and 1969—many (skilled) workers will permanently leave the labor upon reaching retirement age. This is expected to have far-reaching consequences for the economy in Germany and Europe, as relatively few (qualified) employees are entering the labor market due to demographic developments, and the social security systems will come under increasing strain (Bäcker 2018).

Findings from a recent survey in Germany show that 25 percent of employees do not believe they will be able to continue working until the statutory retirement age under their current working conditions: Individuals who describe their current work situation as very or extremely stressful are particularly unlikely to believe they can reach the statutory retirement age (Blank and Brehmer 2023). These results indicate that, for employees, the overall workload and stress associated with work demands are key determinants of retirement timing.

Previous research on the relationship between work demands and preferred retirement age in Germany has rarely considered the cumulative nature of these work demands (Scharn et al. 2018; Browne et al. 2019). Yet in reality, work demands rarely occur in isolation. Instead, workers often face multiple burdens simultaneously, and their combined impact may be more detrimental than individual factors assessed separately. This is a notable gap, as the accumulation, i.e., the number of unfavorable work demands experienced simultaneously or in combination, is perceived as burdensome and may impact employees’ health, employment status, and also their retirement age in the medium to long term (Mänty et al. 2016). Furthermore, accumulation may not only occur at a single point in time, but may also extend across the (entire) course of employment (Trischler and Kistler 2010). This article addresses this research gap by examining how multiple work demands relate to the preferred retirement age, using cross-sectional and longitudinal data.

Previous studies on the relationship between physical, psychosocial, and environmental work demands and retirement (intentions) have yielded mixed results (Böckerman and Ilmakunnas 2020; Dal Bianco et al. 2015; Carr et al. 2016). In terms of physical demands, certain findings are consistent: For instance, unfavorable postures or heavy lifting are associated with early retirement preferences in Germany (d’Errico et al. 2021). Similar results have been found in Denmark, where high physical work demands predict early retirement (Sundstrup et al. 2021). Another Danish study found that working in extreme postures, such as twisting or bending the neck or back, and prolonged standing or squatting significantly increases the likelihood of early retirement (Lund and Villadsen 2005). However, studies from England (2016) and the Netherlands (Wind et al. 2014) suggest that physical work demands have little influence on retirement decisions.

To date, little is known about the role of environmental work demands in shaping preferred retirement age. However, Böckerman and Ilmakunnas (2020) found a greater number of perceived environmental hazards and risks, such as exposure to heat, cold, chemicals, or infectious diseases, which correlate with an increased likelihood of early retirement.

With regard to psychosocial work demands, a systematic review by Browne et al. (2019) found inconsistent results across studies. Nonetheless, several significant correlations were identified, particularly between early retirement intentions and factors such as role conflicts, time pressure, and quantitative and emotional demands (Browne et al. 2019). Carr et al. (2016) come to similar conclusions, finding that high psychosocial demands, especially “working speed” and “time pressure,” are associated with early retirement intentions (Carr et al. 2016).

The heterogeneous results can be attributed to different factors. First, the operationalization of constructs varies across studies. Multi-item scales are often used but may rely on different underlying indicators. Second, studies alternately measure preferred versus actual retirement age, potentially leading to different conclusions (Browne et al. 2019). Third, the associations between work demands and (early) retirement may be masked by strong correlations with other variables, such as subjective health status (Stengård et al. 2023). To avoid over-adjustment through such potential mediators, we follow Stengård et al. 2023) and draw on the concept of “bad controls” (Angrist & Pischke 2009) by excluding health and job satisfaction. In light of these considerations, the present study not only focuses on multiple work demands, but also examines whether multiple work demands show a different association with early retirement preference than single working conditions. This allows us to assess whether associations are driven by specific dimensions or by the combined burden of multiple demands.

As the Baby Boomer generation enters retirement in the near future, the (policy) debate should not be limited to pension legislation alone, but must also explore workplace strategies that encourage extended working life. In order to respond effectively to demographic developments, it is essential to identify work-related factors that discourage continued labor force participation and undermine the overall quality of work (Steiber and Kohli 2017). This study contributes to the literature by integrating both cross-sectional and longitudinal perspectives, thereby capturing not only the multiple burden of work demands at a single point in time but also how changes in these demands influence employees’ retirement preferences. Accordingly, this article investigates the relationship between (multiple) work demands and employees’ preferred retirement age, focusing on the following questions:

1. What is the relationship between (multiple) work demands and preferred retirement age?
2. How does the preferred retirement age change when (multiple) work demands change over time?

Unlike previous studies, this article explicitly focuses on multiple work demands and preferred retirement age and their temporal dynamics. The analyses are based on the “BAuA-Working Time Survey” conducted by the German Federal Institute for Occupational Safety and Health, which allows for both cross-sectional and longitudinal analysis.

## Statutory old-age pension and early retirement options in Germany

Currently, there are three types of old-age pensions in Germany with different requirements, excluding the old-age pension for severely disabled persons. The regular old-age pension requires at least five years of contribution to the statutory pension insurance. Since 2012, the minimum age for the regular old-age pension has been gradually increasing from 65 to 67, depending on the employee’s year of birth. This process will be completed in 2031, meaning that people born in 1964 or later will be able to retire at the age of 67. Early retirement in Germany is possible at the age of 63 via old-age pension for long-term insured persons (35 years of contributions). However, early retirement via this old-age pension is penalized financially, with a permanent reduction in pension payments for each month (0.3 percent) of early retirement. The amount of the reductions depends on the regular retirement age and may increase to up to 14.4 percent as part of the gradual increase to 67 years of the regular old-age pension. Since 2012, there is a second option for early retirement, requiring 45 years of contributions, starting with a minimum age of 63 years. As with the regular old-age pension, the earliest possible age for claiming this pension also increases gradually to 65 years of age (Brussig et al. 2016). According to statistics of the German Pension Insurance from 2023, more than half (55 percent) of employees choose to retire early: About 31 percent used the pension with 45 years of contributions, whereas approximately 24 percent choose the option with penalties. On average, individuals accepted reductions of 8.9 percent in 2023. This corresponds to an earlier retirement of approximately 2.5 years. This trend has been continuing for more than a decade (Deutsche Rentenversicherung Bund 2024).

## Data and methods

### Data source and sample

We draw on data from the German telephone survey “BAuA-Working Time Survey,” a biennial panel study conducted by the Federal Institute for Occupational Safety and Health. The survey uses a dual frame random digit dialing (RDD) approach to contact employees via both landline (70%) and mobile numbers (30%)^1^ (e.g., Häring et al. 2016, 2024). It is designed to be broadly representative of the employed population in Germany. The panel survey covers employees working at least ten hours per week,^2^ including information on, e.g., occupation, working hours and conditions as well as health. The panel comprises five waves with a two-year cycle from 2015 to 2023. For the purpose of the analyses on retirement intentions, we restrict the sample to women and men aged 50 and above in dependent employment, whereby civil servants and self-employed are excluded due to different pension regulations.

For the regression analyses, cases with missing values on relevant variables were excluded. Accordingly, the dataset includes two analytically distinct groups: a cross-sectional sample of respondents with data from a single wave (*n* = 32,686) and a longitudinal sample comprising individuals who participated in at least two survey waves (*n* = 12,541).

While the survey captures only the *preferred* rather than the *actual* retirement age, it offers uniquely detailed information on working conditions that are rarely available in other data sources. This richness makes it particularly valuable for our analysis. In addition, prior research demonstrates that in Germany and other European countries, individuals’ preferred and actual retirement ages are often closely correlated, supporting the use of preferred retirement age as a meaningful proxy (Engstler 2019; Steiber and Kohli 2017; Solem et al. 2016).

###  Measures

The dependent variable for our analyses is the preferred retirement age, which is determined by the question “*If you could decide freely, would you like to retire early, would you like to work until the statutory retirement age, or would you like to work beyond the statutory retirement age?*” For analytical purposes, we created a binary variable: Responses indicating a preference to retire early were coded as 1, while preferences to work until or beyond the statutory retirement age were combined and coded as 0, serving as the reference category. In the 2023 survey wave, the question format was modified, and respondents were asked to provide their preferred retirement age in years. To ensure comparability across waves, we recoded this information into categorical responses aligned with the earlier structure and statutory pension regulations.

The independent variables are the work demands for which the respondents were asked about their frequency of occurrence. The possible answers “never,” “rarely,” and “sometimes” are grouped together as the reference category and compared with the response “frequently.” For the analysis of multiple working conditions, only the answer “frequently” is counted, resulting in a sum score representing the total number of frequently occurring physical and psychosocial work demands. The variables considered are selected based on the job exposure matrix developed by Meyer & Siefer (2021). The physical work demands consist of six items: working in a standing position, lifting/carrying heavy loads (men: 20 kg; women: 10 kg), exposure to cold/heat/moisture/dampness/draughts, work in a bending/squatting/kneeling/recumbent position or overhead, harsh/bad/insufficient lighting, and exposure to noise. The psychosocial work demands consist of seven items including strong deadline or performance pressure, working very quickly, simultaneous performance of work processes, interruption by colleagues, hiding emotions, confronting others’ people’s problems, and demands due to the amount of work or workload. The variable “demands due to amount of work or workload” is categorized into “more over-challenged” vs. “able to cope/more under-challenged.” In addition to the cumulative sum scores, we also consider the individual work demands in the analysis. Comparing multiple with single work demand items helps disentangle whether associations are attributable to specific dimensions or the combined burden of multiple demands, thereby also reducing potential aggregation bias.

In our analysis we include several sociodemographic and employment characteristics as control variables: sex, age (linear and quadratic), region (eastern or western Germany), birth cohort, marital status, employment contract type, occupational sector, actual weekly working hours and educational level. Furthermore, all models include a wave dummy (2015 and 2017 as base) to control for period-specific effects (e.g., COVID-19 pandemic, policy measures). Notably, in 2015, many employees made use of a pension scheme introduced in 2012 that allowed early retirement after 45 years of pension contributions without pension reductions (Deutsche Rentenversicherung Bund 2024). We explicitly exclude subjective health and job satisfaction from the main analysis (Stengård et al. 2023; Angrist and Pischke 2009) as both variables may themselves be affected by work demands and can therefore act as mediators rather than confounders. Nevertheless, we conduct robustness checks that include these variables, which are presented in the Supplementary Material (S1–S4). We also included tables with coefficients for all variables for Tables 2 and 4 with single items (see S5–S6).

### Methods

We perform wave-specific ordinary least squares regression (OLS) with robust standard errors to examine the (cross-sectional) relationship between (multiple) work demands and preferred retirement age. These cross-sectional models provide a descriptive baseline for whether multiple work demands are associated with retirement preferences before exploiting longitudinal variation. In the panel setting, POLS serves as a between-person benchmark capturing differences across individuals, against which RE and FE estimates can be compared. Accordingly, we test whether there is a relationship between multiple work demands and retirement intention, following the rationale of excluding subjective health and job satisfaction as control variables. Secondly, we conduct pooled OLS (POLS), random-effects regression models (RE), and fixed-effects regression models (FE) with standard errors clustered over individuals to estimate longitudinal effects across waves. POLS and RE regression models assume independence between observations and do not account for unobserved heterogeneity, which may affect estimates due to unobserved between-person differences. POLS and RE therefore capture between-person differences in work demands, while FE isolates within-person changes. For this reason, we focus on FE models which control for time-invariant individual characteristics by using within-person variation (Allison 2009; Brüderl 2010). We report POLS, RE, and FE estimates jointly to illustrate differences between these approaches and to capture different sources of variation relevant to the research question. For reference, we conducted a Hausman test which indicated that FE estimates are preferable compared to RE models. However, substantive model choice is motivated also by differences in within- versus between-person variation. Relying on within variation in FE models also yields disadvantages that should be considered, such as less statistical power due to reduced within-person variation, and potentially attenuated coefficients, no estimates for time-invariant variables, and the inability to account for time-varying unobserved heterogeneity. Importantly, and with regard to our research question, FE models do not capture long-term exposure, as persistent high work demands are differenced out.

As the dependent variable—preference for early retirement—is binary, we estimate linear probability models (LPMs) rather than logit or probit models. LPMs offer the advantage of direct interpretability of coefficients as marginal effects, which facilitates comparison across models and time points. Although LPMs can produce heteroscedasticity and predicted probabilities outside the [0,1] range, they often yield similar substantive conclusions to nonlinear models and are widely used in applied panel research (Angrist and Pischke 2009; Mood 2010). To account for potential heteroscedasticity, we apply (panel) robust standard errors (RSE) for all regression models.

## Results

### Descriptive results

Table 1 shows the sample statistics, including the number of observations for each wave. The table shows that the majority of employees express a preference for early retirement, reflecting actual retirement behavior in Germany (Deutsche Rentenversicherung Bund 2024). Between 2015 and 2023, the average number of physical demands ranges from 0.9 to 1.3. Results from Table 1 indicate that, in total, almost 50 percent of employees report no physical work demands. In contrast, psychosocial work demands are reported more frequently: Employees experience multiple simultaneous demands, with values ranging between 2.7 and 3.0 over the observed period. Across all waves, the majority of employees report at least one psychosocial work demand, whereas only 12.2 percent report none.

**Table 1:** Sample statistics (in %)

	Waves					
	2015	2017	2019	2021	2023	Total
N	7,664	4,552	4,807	9,797	5,866	32,686
Preferred retirement age						
(At least) Regular	39.9	37.8	37.5	34.1	29.8	35.8
Early	60.1	62.2	62.5	65.9	70.2	64.2
Physical Work Demands (Ø)	1.3	1.2	1.1	1.0	0.9	1.1
Physical Work Demands (categorical)						
None	44.1	47.6	48.8	52.2	55.7	49.8
1–3	43.7	42.4	42.3	39.4	37.7	40.9
4 and more	12.2	10.0	8.9	8.4	6.6	9.3
Psychosocial Work Demands (Ø)	2.9	3.0	2.9	2.8	2.7	2.9
Psychosocial Work Demands (categorical)						
None	10.8	10.5	12.0	13.0	14.1	12.2
1–3	48.0	46.4	46.8	49.5	51.5	48.7
4 and more	41.2	43.1	41.2	37.5	34.4	39.1
Sex						
Men	48.1	49.3	50.3	50.6	51.4	49.9
Women	51.9	50.7	49.7	49.4	48.6	50.1
Age (Ø)	55.9	56.6	57.1	57.5	58.4	57.1
Education						
Low	3.0	2.5	2.4	2.6	1.8	2.5
Intermediate	49.1	45.7	43.1	42.0	39.2	43.9
High	47.9	51.8	54.5	55.4	59.0	53.6
Region						
East	16.8	15.9	15.6	14.4	13.9	15.3
West	83.2	84.1	84.4	85.6	86.1	84.7
Cohort						
Up to 1957	34.2	27.3	17.0	8.8	6.0	18.1
1958 to 1963	48.0	42.8	41.6	38.1	33.9	40.9
1964 or later	17.8	29.9	41.4	53.0	60.1	41.1
Marital status						
Married/reg. partnership	66.6	65.0	64.9	64.1	63.5	64.8
Single	12.3	13.9	14.2	16.4	18.0	15.0
Divorced	16.9	16.5	16.3	15.4	14.3	15.8
Widowed	4.2	4.5	4.6	4.2	4.2	4.3
Employment contract						
Fixed-term	5.7	5.1	4.5	4.6	5.2	5.0
Permanent	94.3	94.9	95.5	95.4	94.8	95.0
Occupational Sector						
Production	22.6	22.0	22.2	21.6	21.1	21.9
Service	77.4	78.0	77.8	78.4	78.9	78.1
Actual weekly working hours (Ø)	38.3	38.2	37.6	37.6	37.2	37.8

Source: BAuA-Working Time Survey 2015, 2017, 2019, 2021, 2023; unweighted results

Regarding sociodemographic characteristics, most employees reside in western Germany and a majority have an intermediate or high level of education, indicating that the sample underrepresents employees with low educational attainment. This is, however, a common issue in survey data. There are no substantial differences across waves with respect to sex or marital status but cohort composition shifts over time. The oldest cohort becomes proportionally smaller with each successive wave, while the youngest cohort gains increasing representation. The results from Table 1 also show that the majority (at least 94 percent) have a permanent employment contract, work in the service sector, and work at least 37 h per week. There are hardly any differences between the waves.

Longitudinal descriptive statistics regarding retirement intention show continuity as well as change. Across 11,430 transitions (t to t + 1), 53.5% remain committed to early retirement (*n* = 6,113), and 25.5% remain committed to regular/late retirement (*n* = 2,916). In contrast, 9.3% shift from early to regular/late retirement (*n* = 1,060), and 11.7% shift from regular/late to early retirement (*n* = 1,341). Overall, 21.0% change their retirement intention at least once during the observation period.

Work demands also exhibit within-person change over time (t to t + 1). Between waves, 4,626 transitions involve changes in physical work demands of which 2,051 represent increases. The variation is larger for psychosocial work demands with 8,709 transitions, including 3,844 increases. In total, 37.1% of respondents experience within-person changes in at least one multiple work demand dimension. These descriptive patterns indicate that a non-trivial share of older workers experiences within-person changes in both retirement intentions and work demands.

### Cross-sectional results

**Fig. 1:**
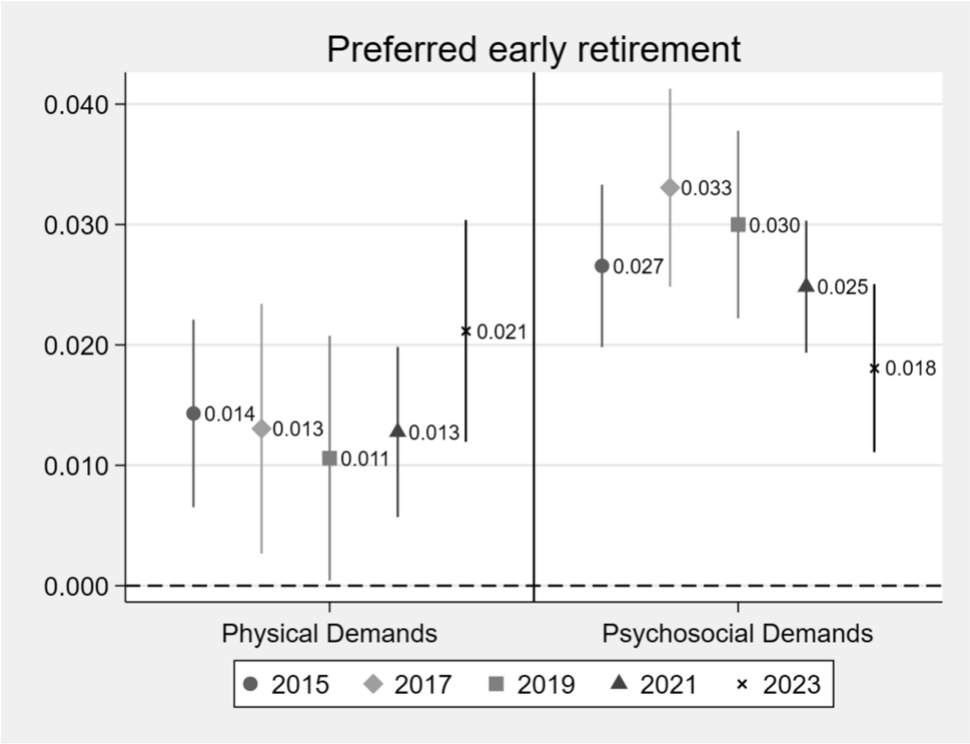
Coefficient plots for the relationship of frequent physical and psychosocial work demands and preferred early retirement from 2015 to 2023

In a first step, we examine the cross-sectional relationship between work demands and preferred early retirement based on multivariate LPM, as illustrated in the coefficient plot in Fig. 1.

Controlled for: Sex, age (linear and quadratic), education, region, cohorts, marital status, employment contract, sector, actual weekly working hours; error bars present; N: 2015: 6,296; 2017: 3,748; 2019: 4,074; 2021: 8,035; 2023: 4,559 Source: BAuA-Working Time Survey 2015, 2017, 2019, 2021, 2023; error bars present

The figure displays statistically significant coefficients in all survey years, indicating that the likelihood of preferring early retirement increases with each additional physical work demand. For example, in 2019 the likelihood increases by 1.1 percentage points and in 2023 by 2.1 percentage points. The coefficients are slightly higher for psychosocial demands, where the probability of preferring early retirement ranges between 1.8 and 3.3 percentage points between waves. The robustness analysis, which includes subjective health status and job satisfaction as control variables (see Table S1, Supplementary Material), yields largely comparable results for psychosocial work demands: Coefficients remain statistically significant across all waves, though slightly attenuated in magnitude. For physical demands, however, only the results for 2023 remain statistically significant after including these additional controls. These results align with the argument put forward by Stengård et al. (2023) that health may act as a mediator or consequence of work demands and that adjusting for health may therefore constitute “bad controls” (Angrist & Pischke 2009) by overcontrolling the relationship between work demands and preference for early retirement.

Table 2 presents the cross-sectional regression analyses, including the single-item specifications of the different physical and psychosocial work demands (frequently vs. sometimes/rarely/never).

**Table 2:** OLS regression models for the relationship between frequent work demands (single items) and preferred retirement timing (2015 – 2023)

	2015		2017		2019		2021		2023	
	Coef	RSE	Coef	RSE	Coef	RSE	Coef	RSE	Coef	RSE
Physical Work Demands										
Working in standing position	− 0.018	0,015	− 0.013	0,018	− 0.027	0,018	− 0.003	0,012	− 0.004	0,016
Kneeling/bending/overhead	0.015	0,019	0.067**	0,026	0.025	0,026	0.013	0,018	0.068**	0,024
Lifting/carrying heavy loads	0.014	0,018	0.006	0,025	0.027	0,024	0.049**	0,017	0.025	0,023
Cold/heat/moisture, etc	− 0.002	0,017	− 0.012	0,024	-0.006	0,022	− 0.003	0,016	− 0.005	0,021
Harsh/insufficient lighting	0.043*	0,020	− 0.007	0,028	0.043	0,025	0.004	0,020	0.081**	0,025
Noise	0.064***	0,016	0.047*	0,020	0.035	0,020	0.026	0,014	0.023	0,018
Psychosocial Work Demands										
Deadline/performance pressure	0.044**	0,015	0.043*	0,019	0.033	0,018	0.022	0,013	0.047**	0,016
Interruption by colleagues	0.036**	0,013	0.021	0,017	0.051**	0,016	0.043***	0,011	0.009	0,014
Simultaneous performance of work processes	− 0.027	0,015	0.024	0,019	− 0.021	0,018	− 0.021	0,013	− 0.009	0,016
Working very quickly	0.017	0,014	0.004	0,019	− 0.002	0,018	0.011	0,012	0.004	0,016
Hiding emotions	0.039*	0,015	0.068***	0,017	0.058***	0,017	0.037**	0,012	0.018	0,017
Confronting other people’s problems	− 0.037**	0,014	− 0.015	0,017	0.008	0,017	0.005	0,012	− 0.016	0,015
Demands due to amount of work or workload	0.135***	0,017	0.120***	0,023	0.129***	0,021	0.101***	0,015	0.073***	0,018
R2	0,07		0,08		0,08		0,10		0,11	
adj. R2	0,06		0,07		0,07		0,10		0,10	
N	6203		3710		4045		7937		4511	

^***^ p < .001, ** p < .01, * p < .05; Controlled for: Sex, age (linear and quadratic), educational level, region, birth cohorts, marital status, employment contract, occupational sector, actual weekly working hours; Source: BAuA-Working Time Survey 2015, 2017, 2019, 2021, 2023

The results show that most coefficients in the category physical work demands (upper panel) are positive, indicating that employees frequently exposed to the respective work demands are more likely to prefer early retirement. The exceptions are working in a standing position and cold/heat/moisture, which have negative coefficients, suggesting that they relate to longer employment. Overall, the associations of the single work demands are relatively small.

With regard to psychosocial work demands (Table 2, lower panel), both positive and negative coefficients appear. Frequent demands such as deadline/performance pressure, interruption by colleagues, hiding emotions, and demands due to the amount of work/workload show positive associations with a preference for early retirement. Other psychosocial demands, such as simultaneous task performance, working quickly, and confronting other’s problems, show inconsistent associations across waves. Robustness analyses suggest that subjective health status and job satisfaction strongly influence these relationships (see Table S2, Supplementary Material).

Taken together, the results from Fig. 1 and Table 2 suggest that multiple exposure to work demands shows a more consistent and pronounced association with early retirement preferences than single items of working conditions. While individual physical or psychosocial demands exhibit varying and sometimes insignificant associations across waves, the combined measure of multiple demands relates more consistently to employees’ retirement preferences. This pattern suggests that the total burden of work demands may better capture the relationship between job stressors and early retirement intentions observed in the data.

### Results from panel-regressions

Table 3 presents the longitudinal results from POLS, RE, and FE regression models examining the association between multiple physical and psychosocial work demands and preferred retirement.

**Table 3:** Multivariate regression models POLS, RE and FE on the relationship between multiple work demands and preferred early retirement

	POLS		RE		FE	
	Coef	RSE	Coef	RSE	Coef	RSE
Physical requirements	0.014***	0,002	0.014***	0,002	0.007	0,005
Psychosocial requirements	0.024***	0,002	0.020***	0,002	0.008*	0,003
Sex (base: Men)						
Women	0.037***	0,008	0.040***	0,008		
Age (linear)	0.191***	0,019	0.177***	0,017	0.196***	0,028
Age (quadratic)	− 0.002***	0,000	-0.002***	0,000	− 0.002***	0,000
Education (base: Low)						
Intermediate	0.069**	0,023	0.058**	0,021	− 0.063	0,105
High	− 0.006	0,023	− 0.012	0,022	− 0.030	0,102
Region (base: East Germany)						
West	− 0.011	0,010	− 0.013	0,009	− 0.021	0,142
Cohort						
1958 to 1963	0.130***	0,011	0.130***	0,012		
1964 or later	0.202***	0,014	0.194***	0,017		
Marital status (base: Married/reg. partnership)						
Single	− 0.030**	0,010	− 0.023*	0,009	0.046	0,037
Divorced	− 0.042***	0,010	− 0.046***	0,009	− 0.045	0,031
Widowed	− 0.010	0,017	− 0.016	0,016	− 0.020	0,056
Employment contract (base: Fixed-term)						
Permanent	0.070***	0,015	0.059***	0,014	0.048*	0,021
Sector (base: Production)						
Service	− 0.041***	0,009	− 0.038***	0,009	− 0.003	0,042
Actual weekly working hours	0.001*	0,000	0.001**	0,000	0.002*	0,001
Period Dummy (base: 2015/2017)						
Waves 2019–2023			0.022**	0,008	− 0.044***	0,012
Intercept	− 5.053***	0,530	− 4.690***	0,487	− 5.454***	0,807
R2	0,06		0.06		0,02	
adj. R2	0,06		0.06		0,01	
N	26,712		26,712		26,712	

^***^ p < .001, ** p < .01, * p < .05; Source: BAuA-Working Time Survey 2015, 2017, 2019, 2021, 2023

The POLS estimates show a positive and significant association for both the number of physical demands and psychosocial demands, while the FE results reveal a significant association only for psychosocial work demands. This indicates that at least the relationship between physical demands and early retirement preference observed in POLS is likely (partly) driven by unobserved, time-invariant individual differences that the FE model accounts for. With respect to psychosocial work demands, the FE model supports that an increase within individuals is associated with a modest but significant increase in the preference for early retirement (0.8 percentage points). The smaller coefficients in the FE models reflect their reliance on within-person changes, which tend to be smaller and less variable than between-person differences captured by POLS or RE. Thus, the FE estimates provide a more conservative and arguably less biased assessment of the longitudinal effect of work demands on retirement preferences. Controlling for health and job satisfaction in sensitivity analyses (see Table S3, Supplementary Material), results for physical and psychosocial work demands are insignificant in the FE but remain significant in the POLS and RE models.

Table 4 shows POLS, RE, and FE regression results for the single-item specification of work demands. The coefficients for physical work demands are mostly significant in the POLS and RE models but become insignificant in the FE models, consistent with the findings of the multiple work demands. This suggests that the observed relationship between physical work demands and preferred early retirement is driven by unobserved heterogeneity or between-person variation.

**Table 4:** Multivariate Regression models POLS, RE and FE on the relationship between work demands (single items) and preferred early retirement

	POLS		RE		FE	
	Coef	RSE	Coef	RSE	Coef	RSE
Physical Work Demands						
Working in standing position	− 0.014	0,008	− 0.015*	0,007	− 0.013	0,014
Kneeling/bending/overhead	0.027*	0,011	0.018	0,010	− 0.007	0,017
Lifting/carrying heavy loads	0.024*	0,010	0.031**	0,010	0.028	0,018
Cold/heat/moisture, etc	− 0.004	0,009	− 0.001	0,009	0.008	0,014
Harsh/insufficient lighting	0.030**	0,011	0.025*	0,010	0.011	0,016
Noise	0.043***	0,009	0.040***	0,008	0.020	0,014
Psychosocial Work Demands						
Deadline/performance pressure	0.036***	0,007	0.027***	0,007	− 0.003	0,010
Interruption by colleagues	0.034***	0,007	0.021***	0,006	− 0.009	0,009
Simultaneous performance of work processes	− 0.013	0,007	− 0.007	0,007	− 0.005	0,010
Working very quickly	0.005	0,007	0.013*	0,007	0.018	0,010
Hiding emotions	0.042***	0,007	0.037***	0,007	0.041***	0,010
Confronting other people’s problems	− 0.011	0,007	− 0.012	0,007	− 0.014	0,011
Demands due to amount of work or workload	0.114***	0,009	0.091***	0,008	0.042***	0,012
Intercept	− 4.933***	0,532	− 4.610***	0,492	− 5.531***	0,813
R2	0,07		0,07		0,02	
adj. R2	0,07		0,07		0,02	
N	26,406		26,406		26,406	

^***^ p < .001, ** p < .01, * p < .05; Controlled for: sex, age (linear and quadratic), educational level, region, birth cohorts, marital status, employment contract, occupational sector, actual weekly working hours; Source: BAuA-Working Time Survey 2015, 2017, 2019, 2021, 2023; see supplementary material (S5 and S6) with all variables included

In contrast, the FE model identifies two psychosocial work demand items with significant coefficients: Frequently hiding emotions is related to an increased likelihood of preferred early retirement by 4.1 percentage points and demands related to the amount of work or workload are associated with an increased probability of preferred early retirement by 4.2 percentage points. Sensitivity analyses controlling for health and job satisfaction (see Table S4, Supplementary Material) yield similar results, indicating that these associations are independent of health and job satisfaction.

## Discussion and conclusions

Discussions about raising the statutory retirement age often fail to consider that there are groups of employees who either do not want to or are not able to remain in employment until reaching the statutory retirement age (Kaboth et al. 2024; Bäcker et al. 2017; Hofäcker 2015). Employees exposed to stressful work demands prefer to retire as early as possible (Blank and Brehmer 2023). Therefore, this article examines the relationship between (multiple) work demands and preferences for early retirement.

The cross-sectional analyses show that both the number of multiple physical and psychosocial work demands significantly relate to early retirement preferences across nearly all survey waves. However, when examining individual work demand as single items, this relationship appears weaker and less consistent, suggesting that it is the combined burden of multiple work demands—rather than single exposures—that shapes retirement timing preferences. The longitudinal analyses using FE models further support this. Increasing number of multiple psychosocial work demands are significantly associated with preferred early retirement even when accounting for time-invariant heterogeneity, though the coefficients are smaller. By contrast, the association between physical demands and retirement preferences seems largely driven by unobserved between-person differences (see POLS and RE models). Although the effects in the FE model are modest, they should not be dismissed. Given that the study’s observation window of eight years with biennial data is limited in capturing long-term trajectories over the full working life, even small effects within this timeframe could accumulate meaningfully across the employment history. It is important to note that FE estimates reflect short- to medium-term within-person change rather than long-term accumulated exposure, as persistent differences are differenced out by the estimator.

The results concerning psychosocial work demands are in line with previous findings (Browne et al. 2019; Carr et al. 2016; Thorsen et al. 2016), but this study adds that an increase in multiple psychosocial work demands is consistently related to preferred early retirement. From an occupational health perspective, this highlights the need for sustainable and supportive working conditions, particularly as mental health conditions have become the leading cause of disability pensions in Germany (Deutsche Rentenversicherung Bund 2024). Given that reintegration into the workforce following disability retirement remains rare (Zink and Brussig 2022), the combined effects of labor shortages and demographic aging may further intensify pressure on the pension system. Therefore, addressing (multiple) psychosocial work demands is crucial, as it may shape both the ability and willingness to remain in employment and influence quality of life in retirement (Andersen et al. 2021).

The lack of associations between physical work demands and early retirement preferences in the longitudinal models may relate to health selection. Individuals facing high physical demands may experience health shocks (i.e., sudden declines in their health) that may force them to leave the workforce early—even without being eligible to receive disability pension (Jones et al. 2020; Lundborg et al. 2015). As these individuals are no longer employed, they are not captured in the sample studied, potentially resulting in a healthy worker effect (Chowdhury et al. 2017). This selection may contribute to weaker observed associations and an underestimation over time. Such survivor and healthy worker effects are likely to be particularly pronounced among physically demanding occupations and may reinforce socioeconomic gradients in retirement preferences.

### Limitations

Several additional limitations must be acknowledged when interpreting the findings of this study. First, the observational window spans only eight years in biennial intervals, capturing a relatively short phase of the overall employment biography. As a result, long-term trends and processes may be underrepresented, particularly for work demands that change gradually over time. Second, the use of FE models only reflects those employees with changing work demands as well as retirement intention. Employees with persistent exposure to demanding working conditions are not represented. Moreover, physical and psychosocial work demands are included simultaneously in the regression models in our study, which allows for mutual adjustment. Future studies could additionally examine whether physical and psychosocial work demands interact or compensate for each other (e.g., within a JD-R framework). In addition, the index used sums concurrent demand dimensions, which may mask heterogeneity across components. Future studies may disentangle specific combinations of exposures. The observation window further encompasses major macro-level disruptions, most notably the COVID-19 pandemic, which affected working conditions and may have altered retirement preferences among older workers including documented increases in psychosocial stress, economic worries and health inequalities in Germany (e.g., Demirer and Pförtner 2023; Demirer et al. 2025; Wahrendorf et al. 2023). While wave dummies capture common period shocks at the macro-level, within-person changes in the FE models may partly reflect such crisis-related adjustments rather than gradual exposure. This should be kept in mind when interpreting the longitudinal estimates. Third, the dependent variable reflects retirement preferences rather than actual retirement behavior. Although previous studies suggest that preferred and actual retirement ages are generally well aligned and that preferred retirement age serves as a reliable proxy for actual behavior in Germany and other European contexts (Engstler 2019; Steiber and Kohli 2017; Solem et al. 2016), differences may still arise due to unforeseen health shocks, financial limitations, or institutional changes. Relatedly, the study relies on self-reported data, which may introduce recall bias or subjective distortions in reporting work demands and retirement preferences. While common in survey-based research, this limitation may affect the accuracy of demand exposure measurement. Fourth, the sample under represents employees with low educational attainment, which are in general more likely to be exposed to high (physical) work demands and constrained in their retirement choices. As a result, the observed associations may underestimate the impact of work demands in more vulnerable labor market segments. In addition to this selection mechanism, survivor effects (or healthy worker effects) may also arise due to the panel nature of the data. Lastly, the empirical models focus on associations rather than causal inference. Despite the use of FE models to account for unobserved time-constant heterogeneity, potential endogeneity remains—particularly regarding time-varying confounders. Heterogeneity by gender and occupational trajectories also represents an important avenue for future research. Such analyses would require distinct modeling strategies, particularly in the German context where gendered employment biographies and East–West differences shape exposures.

## Concluding remarks

Taken together, the findings suggest that multiple (psychosocial) work demands are more important for understanding early retirement preferences than isolated working conditions. Even within a relatively short panel period, an increase in psychosocial demands is associated with an increased desire to retire earlier. This highlights the need for sustainable working conditions, especially against the background that mental health problems have become the leading cause of disability pensions (Deutsche Rentenversicherung Bund 2024) and may further intensify pressure on the pension system.

Internationally, the findings support research emphasizing job quality as a determinant of labor market participation. In contexts where early retirement options are limited and retirement ages increase, the mismatch between preferred and actual retirement age could grow—unless work demands are better managed across the employment biography. While retirement intentions are strong predictors of subsequent behavior, policy inference should remain cautious given institutional, health and financial contingencies at older ages. Policies extending working life should not focus solely on statutory thresholds but consider accumulated strain throughout employees’ working lives.

## Supplementary Information

Below is the link to the electronic supplementary material.

Supplementary file 1.

## Data Availability

The BAuA-Working Time Survey is available upon application to the Federal Institute for Occupational Safety and Health at https://www.baua.de/DE/Forschung/Forschungsdaten/Forschungsdaten_node.html

## Footnotes

1 Wave-specific response rates are approximately 30 percent (for details, see Häring et al. 2016; Häring et al. 2024).

2 Employees with fewer working hours typically hold marginal “mini-job” positions that do not contribute to the social security system and are thus not the focus of this study.
