# Supplementary material for: Multiple work demands and early retirement intention in Germany: A cross-sectional and longitudinal analysis
Source: Eur J Ageing. 2026 Mar 8;23(1):15. doi: 10.1007/s10433-026-00915-y (PMC13065913; doi:10.1007/s10433-026-00915-y)
Supplement: Supplementary file 1 [file 10433_2026_915_MOESM1_ESM.docx]

**Supplementary Material**

**Robustness analysis with subjective Health and Job Satisfaction (cumulative and single items used)**

Table S 1 Robustness Analysis with health and job satisfaction: OLS regression models for the relationship between cumulative work demands and preferred retirement timing (2015 – 2023)

|  | 2015 | | 2017 | | 2019 | | 2021 | | 2023 | |
| --- | --- | --- | --- | --- | --- | --- | --- | --- | --- | --- |
|  | Coef. | RSE | Coef. | RSE | Coef. | RSE | Coef. | RSE | Coef. | RSE |
| Physical Work Demands | 0.008 | 0.004 | 0.007 | 0.005 | 0.003 | 0.005 | 0.006 | 0.004 | 0.015** | 0.005 |
| Psychosocial Work Demands | 0.012*** | 0.004 | 0.020*** | 0.004 | 0.018*** | 0.004 | 0.014*** | 0.003 | 0.008* | 0.004 |
| Sex (base: Men) |  |  |  |  |  |  |  |  |  |  |
| Women | 0.041** | 0.014 | 0.031 | 0.018 | 0.013 | 0.017 | 0.057*** | 0.012 | 0.045** | 0.015 |
| Age (linear) | 0.310*** | 0.051 | 0.313*** | 0.063 | 0.327*** | 0.072 | 0.025 | 0.031 | 0.013 | 0.030 |
| Age (quadratic) | -0.003*** | 0.000 | -0.003*** | 0.001 | -0.003*** | 0.001 | -0.000 | 0.000 | -0.000 | 0.000 |
| Education (base: Low) |  |  |  |  |  |  |  |  |  |  |
| Intermediate | 0.040 | 0.036 | 0.067 | 0.050 | 0.127* | 0.055 | 0.109** | 0.035 | -0.011 | 0.047 |
| High | 0.011 | 0.037 | 0.010 | 0.050 | 0.076 | 0.055 | 0.027 | 0.035 | -0.122* | 0.047 |
| Region (base: East Germany) |  |  |  |  |  |  |  |  |  |  |
| West | -0.015 | 0.016 | -0.006 | 0.020 | 0.024 | 0.020 | -0.016 | 0.014 | -0.004 | 0.018 |
| Cohort |  |  |  |  |  |  |  |  |  |  |
| 1958 to 1963 | -0.065** | 0.024 | -0.118*** | 0.033 | 0.055 | 0.041 | 0.398*** | 0.029 | 0.268*** | 0.048 |
| 1964 or later | 0.004 | 0.042 | -0.119* | 0.055 | 0.100 | 0.057 | 0.392*** | 0.039 | 0.292*** | 0.057 |
| Marital status (base: Married/reg. partnership) |  |  |  |  |  |  |  |  |  |  |
| Single | -0.014 | 0.019 | -0.037 | 0.023 | -0.065** | 0.021 | -0.051*** | 0.014 | -0.086*** | 0.018 |
| Divorced | -0.037* | 0.016 | -0.026 | 0.021 | -0.044* | 0.020 | -0.048*** | 0.014 | -0.068*** | 0.019 |
| Widowed | -0.018 | 0.030 | -0.002 | 0.037 | -0.031 | 0.036 | 0.001 | 0.025 | -0.022 | 0.033 |
| Employment contract (base: Fixed-term) |  |  |  |  |  |  |  |  |  |  |
| Permanent | 0.077** | 0.027 | 0.073* | 0.037 | 0.036 | 0.038 | 0.055* | 0.026 | 0.064* | 0.032 |
| Sector (base: Production) |  |  |  |  |  |  |  |  |  |  |
| Service | -0.055*** | 0.016 | -0.046* | 0.020 | -0.029 | 0.019 | -0.026* | 0.013 | -0.029 | 0.017 |
| Actual weekly working hours | 0.002*** | 0.001 | 0.001 | 0.001 | -0.000 | 0.001 | 0.000 | 0.001 | 0.001 | 0.001 |
| Health | 0.062*** | 0.006 | 0.056*** | 0.009 | 0.052*** | 0.008 | 0.056*** | 0.006 | 0.040*** | 0.007 |
| Job satisfaction | 0.104*** | 0.010 | 0.097*** | 0.013 | 0.103*** | 0.012 | 0.068*** | 0.008 | 0.065*** | 0.011 |
| Intercept | -8.092*** | 1.479 | -7.910*** | 1.858 | -8.850*** | 2.009 | -0.681 | 0.861 | 0.244 | 0.857 |
| R2 | 0.09 |  | 0.10 |  | 0.10 |  | 0.12 |  | 0.12 |  |
| adj. R2 | 0.09 |  | 0.10 |  | 0.09 |  | 0.11 |  | 0.11 |  |
| N | 6284 |  | 3743 |  | 4072 |  | 8028 |  | 4554 |  |
| *** p<.001. ** p<.01. * p<.05; Subjective health (1 very good – 5 very bad); satisfaction (1 very satisfied – 4 not satisfied); Source: BAuA-Working Time Survey 2015. 2017. 2019. 2021. 2023 | | | | | | | | |  |  |

Table S 2 Robustness Analysis with health and job satisfaction: OLS regression models for the relationship between work demands (single items) and preferred retirement timing (2015 – 2023)

|  | 2015 | | 2017 | | 2019 | | 2021 | | 2023 | |
| --- | --- | --- | --- | --- | --- | --- | --- | --- | --- | --- |
|  | Coef. | RSE | Coef. | RSE | Coef. | RSE | Coef. | RSE | Coef. | RSE |
| Working in standing position | -0.014 | 0.014 | -0.012 | 0.018 | -0.023 | 0.018 | -0.004 | 0.012 | -0.004 | 0.016 |
| Kneeling/bending/overhead | 0.016 | 0.019 | 0.064* | 0.025 | 0.022 | 0.026 | 0.010 | 0.018 | 0.066** | 0.024 |
| Lifting/carrying heavy loads | 0.010 | 0.018 | 0.004 | 0.025 | 0.016 | 0.024 | 0.044** | 0.017 | 0.013 | 0.023 |
| Cold/heat/moisture. etc. | -0.014 | 0.017 | -0.016 | 0.023 | -0.010 | 0.022 | -0.010 | 0.016 | -0.016 | 0.021 |
| Harsh/insufficient lighting | 0.030 | 0.020 | -0.027 | 0.027 | 0.030 | 0.025 | -0.009 | 0.020 | 0.078** | 0.025 |
| Noise | 0.045** | 0.015 | 0.032 | 0.020 | 0.017 | 0.020 | 0.012 | 0.014 | 0.015 | 0.018 |
| Deadline/performance pressure | 0.026 | 0.014 | 0.029 | 0.019 | 0.018 | 0.018 | 0.011 | 0.013 | 0.035* | 0.016 |
| Interruption by colleagues | 0.024 | 0.013 | 0.016 | 0.017 | 0.044** | 0.016 | 0.037*** | 0.011 | 0.004 | 0.014 |
| Simultaneous performance of work processes | -0.011 | 0.014 | 0.029 | 0.019 | -0.013 | 0.018 | -0.011 | 0.013 | -0.001 | 0.016 |
| Working very quickly | 0.014 | 0.014 | 0.001 | 0.018 | -0.003 | 0.018 | 0.013 | 0.012 | 0.002 | 0.016 |
| Hiding emotions | 0.007 | 0.015 | 0.042* | 0.017 | 0.032 | 0.017 | 0.014 | 0.012 | -0.000 | 0.017 |
| Confronting other people's problems | -0.033* | 0.013 | -0.013 | 0.017 | 0.010 | 0.017 | 0.004 | 0.011 | -0.014 | 0.015 |
| Demands due to amount of work or workload | 0.072*** | 0.018 | 0.053* | 0.024 | 0.066** | 0.022 | 0.048** | 0.015 | 0.034 | 0.019 |
| Sex (base: Men) |  |  |  |  |  |  |  |  |  |  |
| Women | 0.044** | 0.015 | 0.030 | 0.019 | 0.010 | 0.018 | 0.054*** | 0.012 | 0.049** | 0.015 |
| Age (linear) | 0.321*** | 0.052 | 0.327*** | 0.067 | 0.327*** | 0.072 | 0.025 | 0.031 | 0.015 | 0.030 |
| Age (quadratic) | -0.003*** | 0.000 | -0.003*** | 0.001 | -0.003*** | 0.001 | -0.000 | 0.000 | -0.000 | 0.000 |
| Education (base: Low) |  |  |  |  |  |  |  |  |  |  |
| Intermediate | 0.044 | 0.036 | 0.063 | 0.050 | 0.132* | 0.056 | 0.119*** | 0.036 | -0.010 | 0.048 |
| High | 0.020 | 0.037 | 0.003 | 0.051 | 0.084 | 0.057 | 0.039 | 0.036 | -0.120* | 0.048 |
| Region (base: East Germany) |  |  |  |  |  |  |  |  |  |  |
| West | -0.018 | 0.016 | -0.011 | 0.021 | 0.021 | 0.020 | -0.018 | 0.014 | -0.004 | 0.018 |
| Cohort |  |  |  |  |  |  |  |  |  |  |
| 1958 to 1963 | -0.067** | 0.024 | -0.123*** | 0.033 | 0.059 | 0.041 | 0.393*** | 0.029 | 0.258*** | 0.048 |
| 1964 or later | 0.002 | 0.043 | -0.123* | 0.055 | 0.101 | 0.057 | 0.386*** | 0.039 | 0.281*** | 0.057 |
| Marital status (base: Married/reg. partnership) |  |  |  |  |  |  |  |  |  |  |
| Single | -0.014 | 0.019 | -0.035 | 0.023 | -0.066** | 0.022 | -0.051*** | 0.014 | -0.085*** | 0.018 |
| Divorced | -0.035* | 0.016 | -0.025 | 0.021 | -0.045* | 0.020 | -0.047** | 0.014 | -0.065*** | 0.019 |
| Widowed | -0.017 | 0.030 | -0.007 | 0.037 | -0.025 | 0.036 | -0.001 | 0.025 | -0.013 | 0.033 |
| Employment contract (base: Fixed-term) |  |  |  |  |  |  |  |  |  |  |
| Permanent | 0.065* | 0.028 | 0.079* | 0.038 | 0.028 | 0.037 | 0.055* | 0.026 | 0.068* | 0.033 |
| Sector (base: Production) |  |  |  |  |  |  |  |  |  |  |
| Service | -0.045** | 0.016 | -0.038 | 0.021 | -0.027 | 0.019 | -0.023 | 0.014 | -0.023 | 0.017 |
| Actual weekly working hours | 0.002** | 0.001 | 0.001 | 0.001 | -0.000 | 0.001 | 0.000 | 0.001 | 0.001 | 0.001 |
| Health | 0.058*** | 0.006 | 0.053*** | 0.009 | 0.049*** | 0.008 | 0.054*** | 0.006 | 0.038*** | 0.007 |
| Job satisfaction | 0.093*** | 0.010 | 0.091*** | 0.013 | 0.096*** | 0.012 | 0.063*** | 0.009 | 0.061*** | 0.011 |
| Intercept | -8.347*** | 1.520 | -8.283*** | 1.950 | -8.802*** | 2.012 | -0.661 | 0.855 | 0.186 | 0.856 |
| R2 | 0.10 |  | 0.10 |  | 0.10 |  | 0.12 |  | 0.12 |  |
| adj. R2 | 0.09 |  | 0.10 |  | 0.09 |  | 0.11 |  | 0.11 |  |
| N | 6194 |  | 3706 |  | 4043 |  | 7931 |  | 4506 |  |
| *** p<.001. ** p<.01. * p<.05; Subjective health (1 very good – 5 very bad); satisfaction (1 very satisfied – 4 not satisfied); Source: BAuA-Working Time Survey 2015. 2017. 2019. 2021. 2023 | | | | | | | | | | |

Table S 3 Robustness Analysis with health and job satisfaction: Multivariate Regression models with POLS. RE and FE for the relationship of cumulative work demands and preferred retirement timing

|  | | POLS | | | RE | | FE | |
| --- | --- | --- | --- | --- | --- | --- | --- | --- |
|  | | Coef. | | RSE | Coef. | RSE | Coef. | RSE |
| Physical Work Demands | | 0.007** | | 0.002 | 0.008*** | 0.002 | 0.004 | 0.005 |
| Psychosocial Work Demands | | 0.013*** | | 0.002 | 0.012*** | 0.002 | 0.004 | 0.003 |
| Sex (base: Men) | |  | |  |  |  |  |  |
| Women | | 0.041*** | | 0.008 | 0.042*** | 0.008 |  |  |
| Age (linear) | | 0.177*** | | 0.018 | 0.165*** | 0.017 | 0.195*** | 0.028 |
| Age (quadratic) | | -0.002*** | | 0.000 | -0.001*** | 0.000 | -0.002*** | 0.000 |
| Education (base: Low) | |  | |  |  |  |  |  |
| Intermediate | | 0.078*** | | 0.022 | 0.065** | 0.021 | -0.052 | 0.106 |
| High | | 0.013 | | 0.023 | 0.005 | 0.022 | -0.023 | 0.103 |
| Region (base: East Germany) | |  | |  |  |  |  |  |
| West | | -0.005 | | 0.009 | -0.009 | 0.009 | -0.035 | 0.138 |
| Cohort | |  | |  |  |  |  |  |
| 1958 to 1963 | | 0.130*** | | 0.011 | 0.126*** | 0.012 |  |  |
| 1964 or later | | 0.206*** | | 0.013 | 0.189*** | 0.017 |  |  |
| Marital status (base: Married/reg. partnership) | |  | |  |  |  |  |  |
| Single | | -0.047*** | | 0.010 | -0.037*** | 0.009 | 0.046 | 0.037 |
| Divorced | | -0.048*** | | 0.010 | -0.051*** | 0.009 | -0.045 | 0.031 |
| Widowed | | -0.010 | | 0.017 | -0.016 | 0.016 | -0.016 | 0.055 |
| Employment contract (base: Fixed-term) | |  | |  |  |  |  |  |
| Permanent | | 0.071*** | | 0.015 | 0.060*** | 0.014 | 0.046* | 0.021 |
| Sector (base: Production) | |  | |  |  |  |  |  |
| Service | | -0.036*** | | 0.009 | -0.034*** | 0.008 | -0.008 | 0.043 |
| Actual weekly working hours | | 0.001** | | 0.000 | 0.001*** | 0.000 | 0.002* | 0.001 |
| Health | | 0.055*** | | 0.003 | 0.048*** | 0.003 | 0.021*** | 0.005 |
| Job satisfaction | | 0.087*** | | 0.005 | 0.072*** | 0.005 | 0.042*** | 0.008 |
| Period Dummy (base: 2015/2017) | |  | |  |  |  |  |  |
| Waves 2019-2023 | |  | |  | 0.027*** | 0.008 | -0.042*** | 0.012 |
| Intercept | | -4.911*** | | 0.521 | -4.545*** | 0.485 | -5.494*** | 0.805 |
| R2 | | 0.09 | |  | 0.09 |  | 0.02 |  |
| adj. R2 | | 0.09 | |  | 0.09 |  | 0.02 |  |
| N | | 26681 | |  | 26681 |  | 26681 |  |
|  |  | | *** p<.001. ** p<.01. * p<.05; Subjective health (1 very good – 5 very bad); satisfaction (1 very satisfied – 4 not satisfied); ***; Source: BAuA-Working Time Survey 2015. 2017. 2019. 2021. 2023 | | | | | |

Table S 4 Robustness Analysis with health and job satisfaction: Multivariate Regression models with POLS. RE and FE for the relationship between work demands (single items) and preferred retirement timing

|  | | | POLS | | RE | | FE | |
| --- | --- | --- | --- | --- | --- | --- | --- | --- |
|  | | | Coef. | RSE | Coef. | RSE | Coef. | RSE |
| Working in standing position | | | -0.013 | 0.008 | -0.016* | 0.007 | -0.016 | 0.014 |
| Kneeling/bending/overhead | | | 0.025* | 0.011 | 0.017 | 0.010 | -0.006 | 0.017 |
| Lifting/carrying heavy loads | | | 0.017 | 0.010 | 0.025** | 0.010 | 0.024 | 0.018 |
| Cold/heat/moisture. etc. | | | -0.012 | 0.009 | -0.008 | 0.009 | 0.006 | 0.014 |
| Harsh/insufficient lighting | | | 0.017 | 0.011 | 0.014 | 0.010 | 0.005 | 0.016 |
| Noise | | | 0.028*** | 0.008 | 0.029*** | 0.008 | 0.017 | 0.014 |
| Deadline/performance pressure | | | 0.021** | 0.007 | 0.016* | 0.007 | -0.008 | 0.010 |
| Interruption by colleagues | | | 0.026*** | 0.007 | 0.016** | 0.006 | -0.011 | 0.009 |
| Simultaneous performance of work processes | | | -0.003 | 0.007 | -0.001 | 0.007 | -0.007 | 0.010 |
| Working very quickly | | | 0.004 | 0.007 | 0.012 | 0.007 | 0.017 | 0.010 |
| Hiding emotions | | | 0.017* | 0.007 | 0.019** | 0.007 | 0.036*** | 0.010 |
| Confronting other people's problems | | | -0.010 | 0.007 | -0.012 | 0.006 | -0.015 | 0.011 |
| Demands due to amount of work or workload | | | 0.057*** | 0.009 | 0.049*** | 0.008 | 0.029* | 0.012 |
| Sex (base: Men) | | |  |  |  |  |  |  |
| Women | | | 0.041*** | 0.008 | 0.041*** | 0.008 |  |  |
| Age (linear) | | | 0.176*** | 0.018 | 0.165*** | 0.017 | 0.199*** | 0.028 |
| Age (quadratic) | | | -0.002*** | 0.000 | -0.001*** | 0.000 | -0.002*** | 0.000 |
| Education (base: Low) | | |  |  |  |  |  |  |
| Intermediate | | | 0.082*** | 0.023 | 0.070** | 0.022 | -0.059 | 0.117 |
| High | | | 0.018 | 0.023 | 0.010 | 0.022 | -0.027 | 0.111 |
| Region (base: East Germany) | | |  |  |  |  |  |  |
| West | | | -0.008 | 0.009 | -0.012 | 0.009 | -0.019 | 0.137 |
| Cohort | | |  |  |  |  |  |  |
| 1958 to 1963 | | | 0.128*** | 0.011 | 0.123*** | 0.012 |  |  |
| 1964 or later | | | 0.201*** | 0.013 | 0.184*** | 0.017 |  |  |
| Marital status (base: Married/reg. partnership) | | |  |  |  |  |  |  |
| Single | | | -0.047*** | 0.010 | -0.037*** | 0.009 | 0.044 | 0.037 |
| Divorced | | | -0.047*** | 0.010 | -0.050*** | 0.009 | -0.049 | 0.031 |
| Widowed | | | -0.009 | 0.017 | -0.017 | 0.016 | -0.015 | 0.057 |
| Employment contract (base: Fixed-term) | | |  |  |  |  |  |  |
| Permanent | | | 0.069*** | 0.015 | 0.057*** | 0.014 | 0.043* | 0.021 |
| Sector (base: Production) | | |  |  |  |  |  |  |
| Service | | | -0.030** | 0.009 | -0.029*** | 0.009 | -0.004 | 0.043 |
| Actual weekly working hours | | | 0.001* | 0.000 | 0.001** | 0.000 | 0.002* | 0.001 |
| Health | | | 0.053*** | 0.003 | 0.046*** | 0.003 | 0.021*** | 0.006 |
| Job satisfaction | | | 0.080*** | 0.005 | 0.067*** | 0.005 | 0.040*** | 0.008 |
| Period Dummy (base: 2015/2017) | | |  |  |  |  |  |  |
| Waves 2019-2023 | | |  |  | 0.027*** | 0.008 | -0.040*** | 0.012 |
| Intercept | | | -4.843*** | 0.524 | -4.496*** | 0.490 | -5.566*** | 0.811 |
| R2 | | | 0.09 |  | 0.09 |  | 0.02 |  |
| adj. R2 | | | 0.09 |  | 0.09 |  | 0.02 |  |
| N | | | 26380 |  | 26380 |  | 26380 |  |
|  |  | *** p<.001. ** p<.01. * p<.05; Subjective health (1 very good – 5 very bad); satisfaction (1 very satisfied – 4 not satisfied); Source: BAuA-Working Time Survey 2015. 2017. 2019. 2021. 2023; | | | | | | |

**Full Model Table without subjective Health and Job Satisfaction (single items only)**

Table S 5 OLS regression models for the relationship between frequent work demands (single items) and preferred retirement timing (2015 – 2023); All variables included (table 2)

|  | 2015 | | 2017 | | 2019 | | 2021 | | 2023 | |
| --- | --- | --- | --- | --- | --- | --- | --- | --- | --- | --- |
|  | Coef. | RSE | Coef. | RSE | Coef. | RSE | Coef. | RSE | Coef. | RSE |
| Physical Work Demands |  |  |  |  |  |  |  |  |  |  |
| Working in standing position | -0.018 | 0.015 | -0.013 | 0.018 | -0.027 | 0.018 | -0.003 | 0.012 | -0.004 | 0.016 |
| Kneeling/bending/overhead | 0.015 | 0.019 | 0.067** | 0.026 | 0.025 | 0.026 | 0.013 | 0.018 | 0.068** | 0.024 |
| Lifting/carrying heavy loads | 0.014 | 0.018 | 0.006 | 0.025 | 0.027 | 0.024 | 0.049** | 0.017 | 0.025 | 0.023 |
| Cold/heat/moisture. etc. | -0.002 | 0.017 | -0.012 | 0.024 | -0.006 | 0.022 | -0.003 | 0.016 | -0.005 | 0.021 |
| Harsh/insufficient lighting | 0.043* | 0.020 | -0.007 | 0.028 | 0.043 | 0.025 | 0.004 | 0.020 | 0.081** | 0.025 |
| Noise | 0.064*** | 0.016 | 0.047* | 0.020 | 0.035 | 0.020 | 0.026 | 0.014 | 0.023 | 0.018 |
| Psychosocial Work Demands |  |  |  |  |  |  |  |  |  |  |
| Deadline/performance pressure | 0.044** | 0.015 | 0.043* | 0.019 | 0.033 | 0.018 | 0.022 | 0.013 | 0.047** | 0.016 |
| Interruption by colleagues | 0.036** | 0.013 | 0.021 | 0.017 | 0.051** | 0.016 | 0.043*** | 0.011 | 0.009 | 0.014 |
| Simultaneous performance of work processes | -0.027 | 0.015 | 0.024 | 0.019 | -0.021 | 0.018 | -0.021 | 0.013 | -0.009 | 0.016 |
| Working very quickly | 0.017 | 0.014 | 0.004 | 0.019 | -0.002 | 0.018 | 0.011 | 0.012 | 0.004 | 0.016 |
| Hiding emotions | 0.039* | 0.015 | 0.068*** | 0.017 | 0.058*** | 0.017 | 0.037** | 0.012 | 0.018 | 0.017 |
| Confronting other people's problems | -0.037** | 0.014 | -0.015 | 0.017 | 0.008 | 0.017 | 0.005 | 0.012 | -0.016 | 0.015 |
| Demands due to amount of work or workload | 0.135*** | 0.017 | 0.120*** | 0.023 | 0.129*** | 0.021 | 0.101*** | 0.015 | 0.073*** | 0.018 |
| Sociodemographic & Employment |  |  |  |  |  |  |  |  |  |  |
| Sex (base: Men) |  |  |  |  |  |  |  |  |  |  |
| Women | 0.037* | 0.015 | 0.024 | 0.019 | 0.005 | 0.018 | 0.051*** | 0.012 | 0.052*** | 0.015 |
| Age (linear) | 0.343*** | 0.054 | 0.350*** | 0.067 | 0.338*** | 0.073 | 0.034 | 0.031 | 0.022 | 0.030 |
| Age (quadratic) | -0.003*** | 0.000 | -0.003*** | 0.001 | -0.003*** | 0.001 | -0.000 | 0.000 | -0.000 | 0.000 |
| Education (base: Low) |  |  |  |  |  |  |  |  |  |  |
| Intermediate | 0.035 | 0.036 | 0.043 | 0.051 | 0.123* | 0.057 | 0.124*** | 0.036 | -0.011 | 0.048 |
| High | 0.009 | 0.037 | -0.024 | 0.051 | 0.068 | 0.057 | 0.035 | 0.036 | -0.126** | 0.048 |
| Region (base: East Germany) |  |  |  |  |  |  |  |  |  |  |
| West | -0.027 | 0.016 | -0.019 | 0.021 | 0.013 | 0.020 | -0.021 | 0.014 | -0.007 | 0.019 |
| Cohort |  |  |  |  |  |  |  |  |  |  |
| 1958 to 1963 | -0.074** | 0.025 | -0.126*** | 0.034 | 0.065 | 0.042 | 0.403*** | 0.029 | 0.276*** | 0.048 |
| 1964 or later | -0.001 | 0.043 | -0.124* | 0.056 | 0.107 | 0.058 | 0.398*** | 0.039 | 0.300*** | 0.057 |
| Marital status (base: Married/reg. partnership) |  |  |  |  |  |  |  |  |  |  |
| Single | 0.001 | 0.019 | -0.014 | 0.023 | -0.053* | 0.022 | -0.042** | 0.015 | -0.075*** | 0.018 |
| Divorced | -0.027 | 0.016 | -0.016 | 0.021 | -0.042* | 0.020 | -0.043** | 0.015 | -0.063** | 0.019 |
| Widowed | -0.016 | 0.031 | -0.001 | 0.038 | -0.025 | 0.036 | -0.007 | 0.025 | -0.016 | 0.034 |
| Employment contract (base: Fixed-term) |  |  |  |  |  |  |  |  |  |  |
| Permanent | 0.052 | 0.028 | 0.091* | 0.039 | 0.019 | 0.037 | 0.063* | 0.026 | 0.067* | 0.033 |
| Sector (base: Production) |  |  |  |  |  |  |  |  |  |  |
| Service | -0.045** | 0.016 | -0.044* | 0.021 | -0.032 | 0.020 | -0.025 | 0.014 | -0.028 | 0.017 |
| Actual weekly working hours | 0.002* | 0.001 | 0.001 | 0.001 | -0.000 | 0.001 | 0.000 | 0.001 | 0.000 | 0.001 |
| Intercept | -8.654*** | 1.569 | -8.652*** | 1.950 | -8.860*** | 2.023 | -0.745 | 0.875 | 0.140 | 0.852 |
| R2 | 0.07 |  | 0.08 |  | 0.08 |  | 0.10 |  | 0.11 |  |
| adj. R2 | 0.06 |  | 0.07 |  | 0.07 |  | 0.10 |  | 0.10 |  |
| N | 6203 |  | 3710 |  | 4045 |  | 7937 |  | 4511 |  |
| *** p<.001. ** p<.01. * p<.05; Source: BAuA-Working Time Survey 2015. 2017. 2019. 2021. 2023 | | | | | | | | | | |

Table S 6 Multivariate Regression models POLS. RE and FE on the relationship between work demands (single items) and preferred early retirement; All variables included (table 4)

|  | POLS | | RE | | | FE | |
| --- | --- | --- | --- | --- | --- | --- | --- |
|  | Coef. | RSE | | Coef. | RSE | Coef. | RSE |
| Physical Work Demands |  |  | |  |  |  |  |
| Working in standing position | -0.014 | 0.008 | | -0.015* | 0.007 | -0.013 | 0.014 |
| Kneeling/bending/overhead | 0.027* | 0.011 | | 0.018 | 0.010 | -0.007 | 0.017 |
| Lifting/carrying heavy loads | 0.024* | 0.010 | | 0.031** | 0.010 | 0.028 | 0.018 |
| Cold/heat/moisture. etc. | -0.004 | 0.009 | | -0.001 | 0.009 | 0.008 | 0.014 |
| Harsh/insufficient lighting | 0.030** | 0.011 | | 0.025* | 0.010 | 0.011 | 0.016 |
| Noise | 0.043*** | 0.009 | | 0.040*** | 0.008 | 0.020 | 0.014 |
| Psychosocial Work Demands |  |  | |  |  |  |  |
| Deadline/performance pressure | 0.036*** | 0.007 | | 0.027*** | 0.007 | -0.003 | 0.010 |
| Interruption by colleagues | 0.034*** | 0.007 | | 0.021*** | 0.006 | -0.009 | 0.009 |
| Simultaneous performance of work processes | -0.013 | 0.007 | | -0.007 | 0.007 | -0.005 | 0.010 |
| Working very quickly | 0.005 | 0.007 | | 0.013* | 0.007 | 0.018 | 0.010 |
| Hiding emotions | 0.042*** | 0.007 | | 0.037*** | 0.007 | 0.041*** | 0.010 |
| Confronting other people's problems | -0.011 | 0.007 | | -0.012 | 0.007 | -0.014 | 0.011 |
| Demands due to amount of work or workload | 0.114*** | 0.009 | | 0.091*** | 0.008 | 0.042*** | 0.012 |
| Sociodemographic & Employment |  |  | |  |  |  |  |
| Sex (base: Men) |  |  | |  |  |  |  |
| Women | 0.038*** | 0.008 | | 0.040*** | 0.008 |  |  |
| Age (linear) | 0.188*** | 0.019 | | 0.176*** | 0.017 | 0.201*** | 0.028 |
| Age (quadratic) | -0.002*** | 0.000 | | -0.002*** | 0.000 | -0.002*** | 0.000 |
| Education (base: Low) |  |  | |  |  |  |  |
| Intermediate | 0.077*** | 0.023 | | 0.065** | 0.022 | -0.068 | 0.116 |
| High | 0.005 | 0.023 | | -0.002 | 0.022 | -0.031 | 0.110 |
| Region (base: East Germany) |  |  | |  |  |  |  |
| West | -0.013 | 0.010 | | -0.016 | 0.009 | -0.005 | 0.139 |
| Cohort |  |  | |  |  |  |  |
| 1958 to 1963 | 0.126*** | 0.011 | | 0.126*** | 0.012 |  |  |
| 1964 or later | 0.196*** | 0.014 | | 0.187*** | 0.017 |  |  |
| Marital status (base: Married/reg. partnership) |  |  | |  |  |  |  |
| Single | -0.034*** | 0.010 | | -0.026** | 0.009 | 0.043 | 0.037 |
| Divorced | -0.042*** | 0.010 | | -0.045*** | 0.009 | -0.049 | 0.031 |
| Widowed | -0.010 | 0.017 | | -0.018 | 0.016 | -0.018 | 0.058 |
| Employment contract (base: Fixed-term) |  |  | |  |  |  |  |
| Permanent | 0.068*** | 0.016 | | 0.057*** | 0.014 | 0.044* | 0.021 |
| Sector (base: Production) |  |  | |  |  |  |  |
| Service | -0.033*** | 0.009 | | -0.031*** | 0.009 | 0.001 | 0.042 |
| Actual weekly working hours | 0.001 | 0.000 | | 0.001* | 0.000 | 0.002* | 0.001 |
| Period Dummy (base: 2015/2017) |  |  | |  |  |  |  |
| Period 2019-2023 |  |  | | 0.023** | 0.008 | -0.041*** | 0.012 |
| Intercept | -4.933*** | 0.532 | | -4.610*** | 0.492 | -5.531*** | 0.813 |
| R2 | 0.07 |  | | 0.07 |  | 0.02 |  |
| adj. R2 | 0.07 |  | | 0.07 |  | 0.02 |  |
| N | 26406 |  | | 26406 |  | 26406 |  |
| *** p<.001. ** p<.01. * p<.05; Source: BAuA-Working Time Survey 2015. 2017. 2019. 2021. 2023 | | | | | | | |
